# Brigade Surgeon in the Army

**Published:** 1862-08

**Authors:** 


Brigade Surgeon in the Army.—The entire Medical Department of the Army has been so thoroughly remodeled by recent congressional enactments, that it requires the attentive brain to keep regularly the run of things. A recent act, however, has at one swoop abolished the entire rank of Brigade Surgeon; and this class of medical officers are now subject to the same rules which govern surgeons—*i. e.*, they cease to exercise a purveyorship, and are liable to active duty as surgeons proper. We notice, too, that we have been occupying to some extent the position of Brigade Surgeons, Medical Inspectors. Many of these new appointees are taken from the old corps of Brigade Surgeons, though we do not understand this to be necessarily so, Among the recent appointments we are pleased to notice that Dr. W. H. Mussey, of our city, late a Brigade Surgeon, is made a Medical Inspector in General Halleck's Department. The corps of surgeons is also to be enlarged by the appointment of one hundred and sixty more for the war; forty being full surgeons, and the remainder assistant surgeons.—*Cincinnati Lancet and Observer.*

